# Supplementary material for: Correlative single-cell hard X-ray computed tomography and X-ray fluorescence imaging
Source: Commun Biol. 2024 Mar 7;7:280. doi: 10.1038/s42003-024-05950-y (PMC10917812; doi:10.1038/s42003-024-05950-y)
Supplement: Supplementary file 2 — Supplemental information file [file 42003_2024_5950_MOESM2_ESM.pdf]

Supplementary information

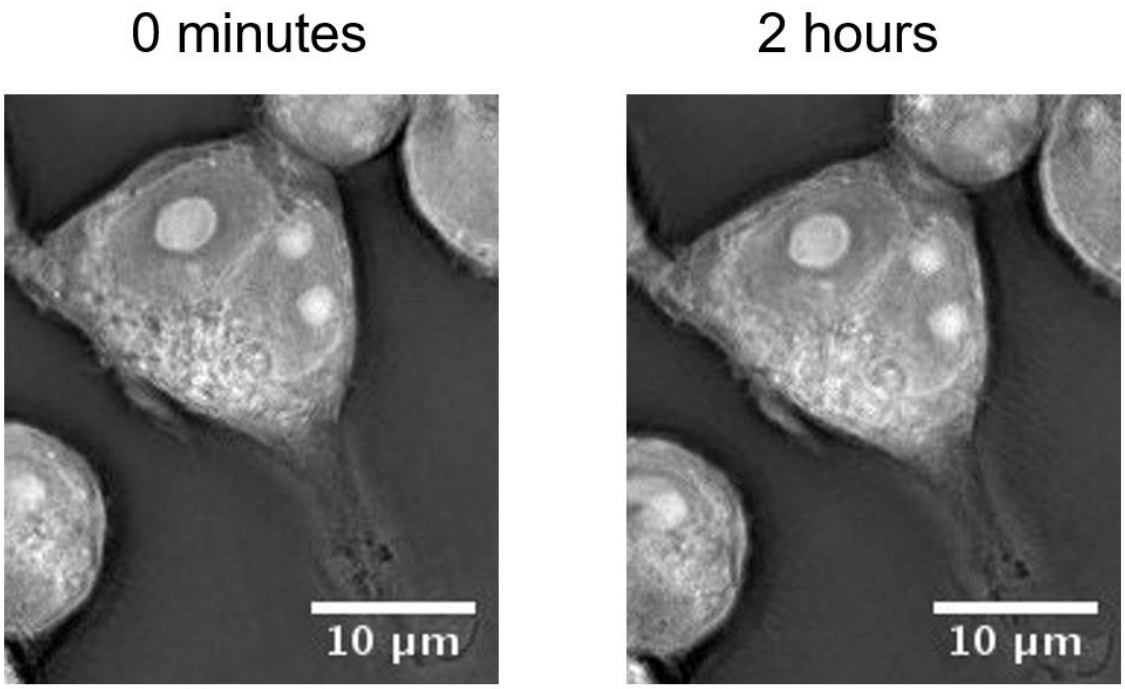

Supplementary Fig. 1. Nanolive live-cell imaging to monitor the PFA fixation process. The first and the last images of fixation imaging were selected to investigate the feature changes caused by the fixative. Some minor changes were observed such as the plasma membrane shrinkage on the top of the cell and the faded nucleus membrane. Although the fixative damage occurred, the overall cell structure didn't have a significant change in the 2-hour fixation.

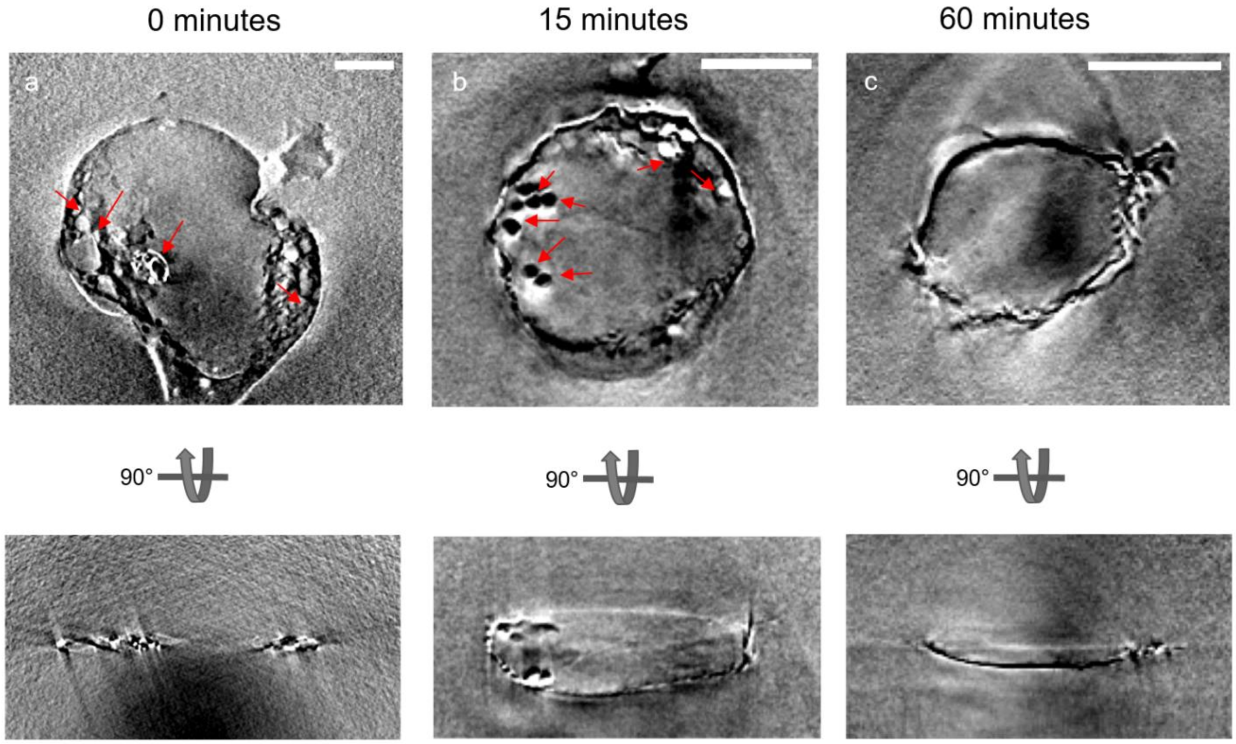

Supplementary Fig. 2. XCT reconstructions of insufficiently fixed cells. The projections of XCT tomograms at two orthogonal views. Arrows indicate bubbles induced by X-ray exposure. Scale bar is 5 μm. **a,** no fixation (0 minutes)**. b,** 15 minutes of fixation. **c,** 60 minutes of fixation.

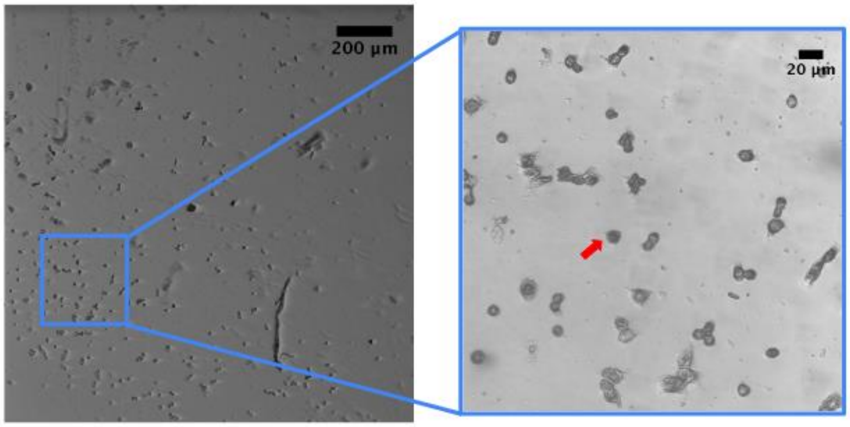

Supplementary Fig. 3. Multi-resolution confocal images for targeting and labeling cells for correlative multimodality X-ray imaging data collection. Both images played important roles in displaying the target cell location that provided a reference for X-ray imaging. A red arrow indicates the cell used for this study.

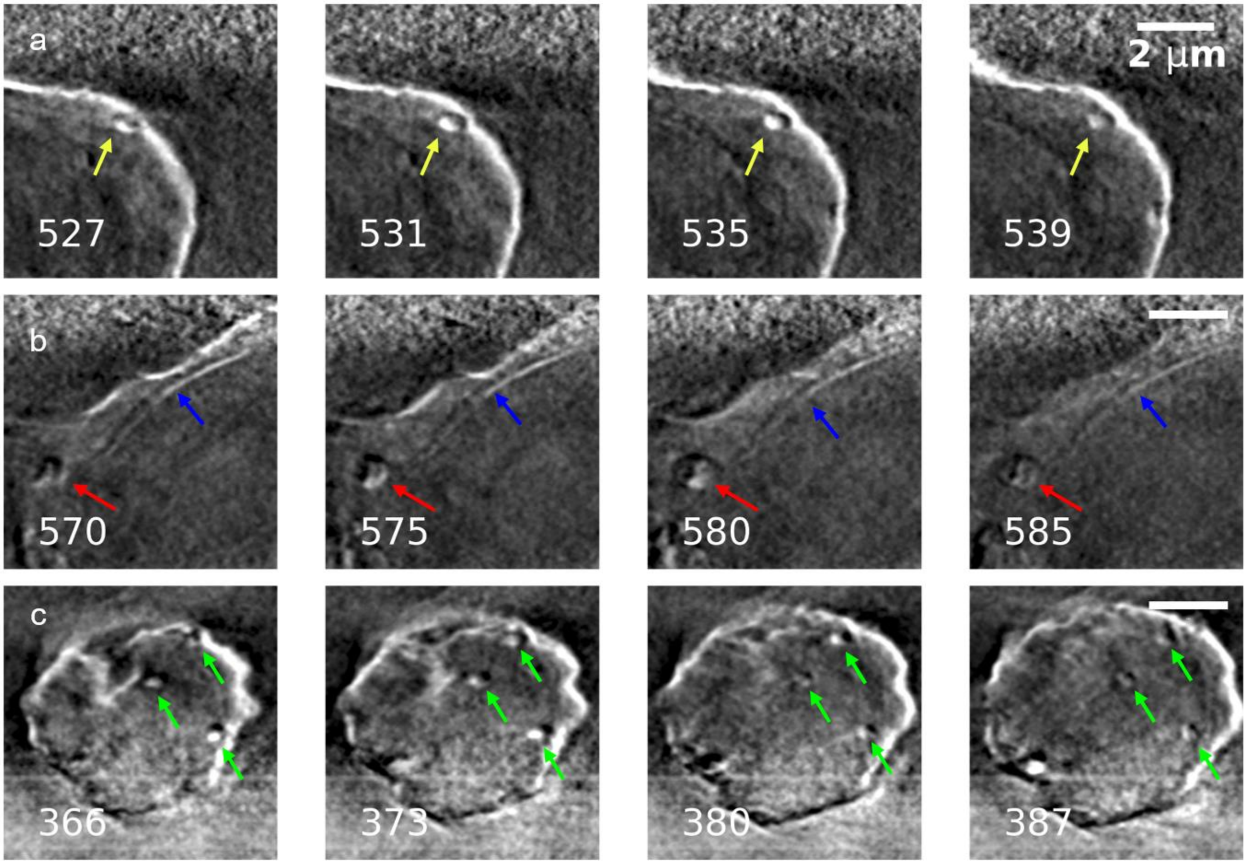

Supplementary Fig. 4. Representative slice views of intracellular organelles. Lysosome (**a**, yellow), mitochondria (**b**, red), nucleus membrane (**b**, blue), and lipid droplets (**c**, green) were observed in the reconstructed tomogram. Scale bars are 2 µm. Numbers at the bottom left of each slice indicate the slice number in the 3D reconstructed tomogram. Each slice is 21.7 nm in thickness. Distances between the first slice and fourth slice for **a**, **b,** and **c** are 0.26 µm, 0.33 µm, and 0.46 µm, respectively.

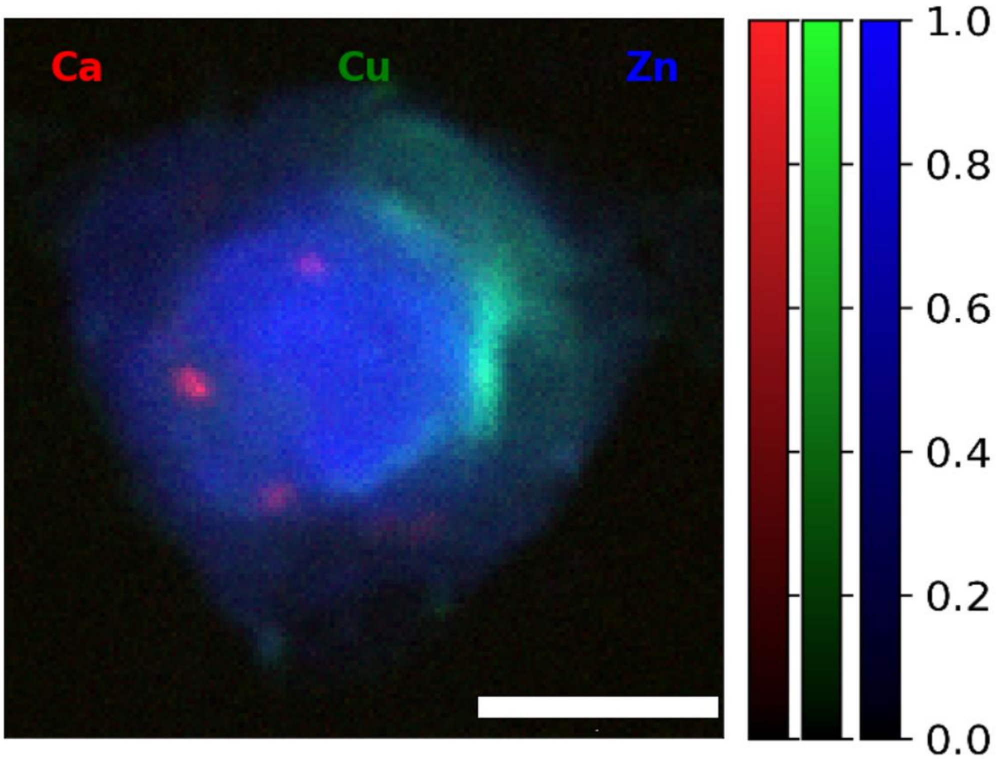

Supplementary Fig. 5. X-ray fluorescence imaging indicates relative localization of Ca, Cu, and Zn. Intensities are normalized for each component to showcase the relative amount in the cell. Scale bar is 5 μm.

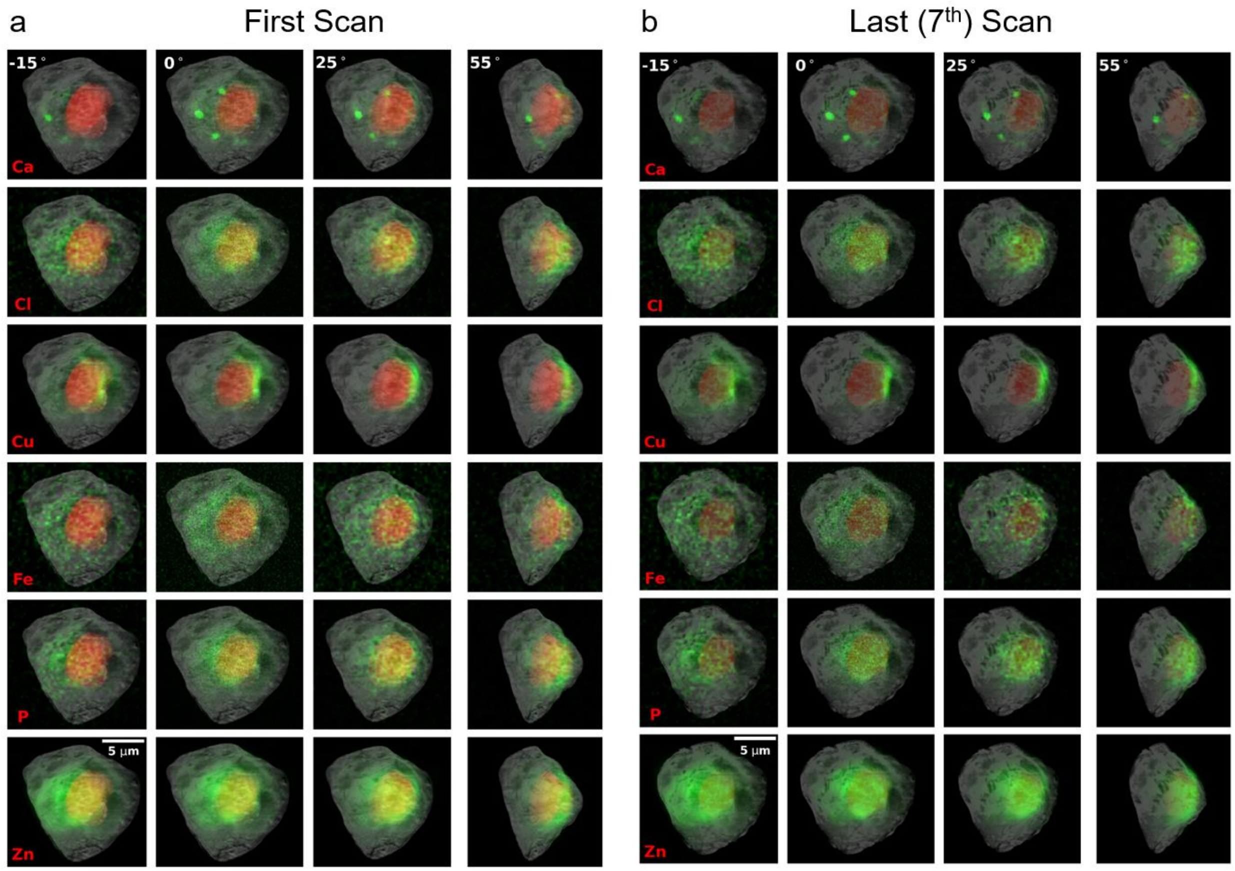

Supplementary Fig. 6. Correlation of multi-modality data. Superimposition of single-cell 2D XRF with the first (**a**) and last (**b**) scans of the 3D XCT images along 4 different angles. The cell was rotated along the y-axis by -15°, 0°, 25°, and 55° to collect angular XRF data. Gray scale maps show the max projection of 3D XCT of the cells. Red masks show segmented nucleus from the 3D XCT images. Green maps show the corresponding localization of indicated chemical elements.

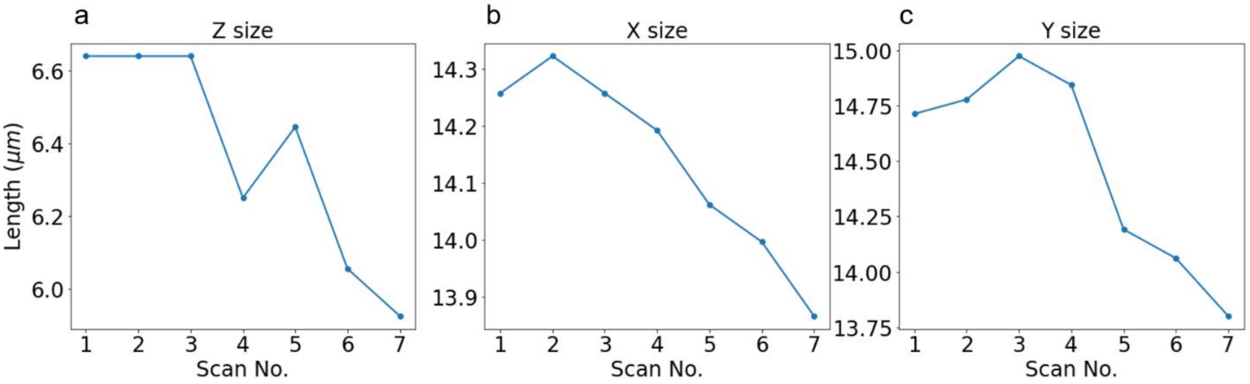

Supplementary Fig. 7. Change of cell size caused by radiation damage*.* The size of the cell was measured in three orthogonal directions, namely, the Z, X, and Y axes, using the maximum length as the basis with respect to the number of XCT scans. Panels **a-c** illustrate the changes in size across seven different scans along the Z, X, and Y axes.
