# Supplementary material for: Correlative single-cell hard X-ray computed tomography and X-ray fluorescence imaging
Source: Commun Biol. 2024 Mar 7;7:280. doi: 10.1038/s42003-024-05950-y (PMC10917812; doi:10.1038/s42003-024-05950-y)
Supplement: Supplementary file 3 — Description of Additional Supplementary Files [file 42003_2024_5950_MOESM3_ESM.pdf]

Description of Additional Supplementary Files

File name: Supplementary Movie 1

Description: Live cell imaging to monitor the PFA fixation process

File name: Supplementary Movie 2

Description: Sliced view of X-ray computed tomography (XCT) from top of the cell to the bottom membrane support.
