# Supplementary figures and images for: Correlative single-cell hard X-ray computed tomography and X-ray fluorescence imaging
Source: Commun Biol. 2024 Mar 7;7:280. doi: 10.1038/s42003-024-05950-y (PMC10917812; doi:10.1038/s42003-024-05950-y)

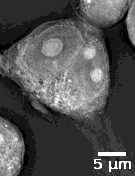

Supplement: Supplementary file 4 — Supplementary Movie 1 [file 42003_2024_5950_MOESM4_ESM.gif]

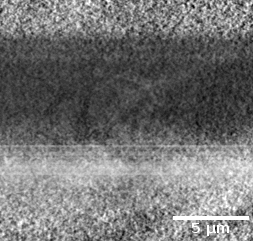

Supplement: Supplementary file 5 — Supplementary Movie 2 [file 42003_2024_5950_MOESM5_ESM.gif]
